# Older Adult Sexuality, Partnership, and Health: Cohort Comparisons of Baby Boomers and Traditionalists

**DOI:** 10.1093/geroni/igaa057.1612

**Published:** 2020-12-16

**Authors:** Winnie Tong, Linda Waite

**Affiliations:** University of Chicago, Chicago, Illinois, United States

## Abstract

This paper updates prior work on older adult sexuality, partnership, and health by examining the most current wave of the National Social Life, Health, and Aging Project (2015-16), a population-based study of health and social factors on a national scale. Comparing data from Wave I, Cohort 1 (2005-06) and Wave I, Cohort 2 (2015-16), we ask whether there are differences in partnership, sexual behaviors and health outcomes between two cohorts (‘Traditionalists’ vs. ‘Baby Boomers’). Additionally, we examine whether sexual frequency is related to physical health, particularly the health conditions of arthritis, diabetes, cognitive impairment, and prior stroke, in both cohorts. We find significant differences between cohorts through a logistic model. For Traditionalists, age, gender, education level, partnership status and diabetes were all significantly related to sexual activity (p < 0.001). Older adults were less sexually active; men were more sexually active; the higher educated were more sexually active; diabetes patients were less sexually active; and partnered were more sexually active. For Baby Boomers, only age and partnership status were significantly related to sexual activity (p < 0.001); gender and diabetes diagnosis were also related (p <0.005). Significantly, partnership status for Boomers is negatively related to sexual activity; the other three relationships – age is related to less sexual activity, men have slightly higher sexual activity, and diabetes was related to less sexual activity – were as expected. Importantly, our findings may imply that partnership or marriage is not as significant to sexual activity, or to health outcomes, as previously believed.

